# The Great Imitator Behind a Rare Mask: Secondary Syphilis Diagnosed During an Atypical Flare of Tumoral Calcinosis

**DOI:** 10.7759/cureus.113054

**Published:** 2026-07-20

**Authors:** Eric Walker, Seema J Ranabhat, Anuva Ray

**Affiliations:** 1 Internal Medicine, Cape Fear Valley Medical Center, Fayetteville, USA

**Keywords:** anchoring bias, diagnostic overshadowing, ectopic calcification, syphilis, treponema pallidum, tumoral calcinosis

## Abstract

Tumoral calcinosis is a rare disorder of ectopic calcium phosphate deposition, usually involving periarticular soft tissues around large joints. Depending on etiology, it may be associated with hyperphosphatemia, fibroblast growth factor 23 pathway abnormalities, recurrent inflammation, ulceration, secondary infection, and progressive functional limitation. Syphilis, caused by *Treponema pallidum*, remains one of the classic infectious mimics in clinical medicine. Secondary syphilis may present with constitutional symptoms, rash involving the palms and soles, arthralgia, periostitis, tenosynovitis, and inflammatory arthritis, thereby overlapping with the presentation of rheumatic and metabolic bone diseases. We present a case of a 37-year-old man with longstanding nodular tumoral calcinosis who presented with fatigue, generalized arthralgia, worsening discomfort at established calcification sites, and painless palmar lesions. Because of his prior history of tumoral calcinosis-associated ulceration and superficial infection, the initial clinical frame favored a typical tumoral calcinosis flare. Serologic testing, performed because of systemic symptoms and unusual palm lesions, revealed a positive Rapid Plasma Reagin (RPR) and titer of 1:32 with positive *Treponema pallidum* antibody. Directed sexual history elicited recent unprotected exposures. The patient was diagnosed with secondary syphilis transitioning into early latent syphilis and was treated with doxycycline 100 mg twice daily for 14 days as Penicillin G Benzathine was not available because of supply shortage. This case report highlights diagnostic overshadowing, anchoring bias, and the need to preserve a broad infectious differential when patients with established rheumatologic or metabolic disease develop atypical systemic features.

## Introduction

Tumoral calcinosis is an uncommon clinicopathologic entity characterized by lobulated deposits of calcium phosphate in extraosseous soft tissues, most often adjacent to large joints such as the hip, shoulder, elbow, and knee. The term is often used descriptively, but clinically meaningful subgroups include hyperphosphatemic familial tumoral calcinosis, normophosphatemic familial tumoral calcinosis, secondary tumoral calcinosis related to chronic kidney disease or disordered mineral metabolism, and idiopathic or sporadic forms. Hyperphosphatemic familial tumor calcinosis reflects impaired fibroblast growth factor 23 (FGF23) signaling through pathogenic variants in FGF23, N-acetylgalactosaminyltransferase 3 (GALNT3), or Klotho gene (KL), leading to elevated serum phosphate, increased calcium-phosphate product, and progressive periarticular calcification [1]. Normophosphatemic familial tumoral calcinosis in contrast is caused by mutation in sterile alpha motif domain-containing protein 9 (SAMD9) gene. The mechanism is incompletely understood but is thought to involve loss of SAMD9's tumor-suppressor and anti-inflammatory functions, permitting dystrophic calcification through unchecked local inflammation [2].

For rheumatologists, tumoral calcinosis sits at the intersection of metabolic bone disease, soft-tissue mass evaluation, crystal deposition, chronic inflammation, and infection risk. Imaging can be striking, often showing multilobulated, cloud-like or cystic calcific masses with internal fluid-calcium levels. Because lesions may be large, tumor-like, ulcerated, or recurrent, patients frequently undergo repeated imaging, drainage procedures, biopsies, orthopedic evaluations, and infectious workups. Over time, both patient and clinician may develop a stable explanatory model in which new pain, skin breakdown, or inflammatory symptoms are attributed to the known calcinosis. That model is often useful, but it can become dangerous when it prevents recognition of a second diagnosis [3-5].

Syphilis is a systemic infection caused by *Treponema pallidum* and remains clinically relevant despite long-standing availability of effective therapy. The infection classically progresses through primary, secondary, latent, and tertiary stages, although the boundaries between stages may be clinically blurred. Secondary syphilis is particularly important to rheumatology because it can present with fever, malaise, weight loss, lymphadenopathy, rash, mucous patches, alopecia, hepatitis, nephropathy, ocular disease, and musculoskeletal manifestations. Reported musculoskeletal features include arthralgia, inflammatory arthritis, tenosynovitis, bursitis, periostitis, osteitis, myositis, and destructive-appearing lesions that may mimic inflammatory rheumatic disease; malignancy; synovitis, acne, pustulosis, hyperostosis, osteitis (SAPHO) syndrome; chronic recurrent multifocal osteomyelitis; septic arthritis; or seronegative spondyloarthritis. Gummatous secondary or tertiary lesions and nodular treponemal deposits have also been described as tumor-like masses on imaging and examination [6].

This case report describes a patient with longstanding tumoral calcinosis in whom secondary syphilis was initially obscured by the chronic metabolic diagnosis. While syphilis is a recognized mimic of tumor-like soft tissue and osseous lesions, to our knowledge, no prior report has described secondary syphilis presenting concurrently with, and masked by, a presumed flare of tumoral calcinosis in a patient with an established diagnosis. The case is not presented merely as an unusual coexistence of two conditions. Its central teaching value is cognitive and practical: in patients with rare chronic disease, clinicians must actively separate expected disease behavior from atypical features, especially when constitutional symptoms, palmar or plantar lesions, sexual exposure risk, or unexplained inflammatory findings are present. The case also provides a framework for evaluating infectious mimics in rheumatology before escalating anti-inflammatory or immunosuppressive treatment.

## Case presentation

A 37-year-old man with nodular tumoral calcinosis diagnosed in 2006 presented for evaluation of three weeks of progressive fatigue, generalized arthralgia, and increased discomfort at several previously known calcification sites. He described a familiar background of intermittent local pain related to calcified masses. He also reported reduced energy and increased generalized stiffness without a clear mechanical trigger. He denied recent trauma. There was no history of chronic kidney disease, known autoimmune rheumatic disease, inflammatory bowel disease, psoriasis, or chronic immunosuppressive therapy.

His tumoral calcinosis history was clinically significant. Genetic testing was ordered to evaluate FGF23, GALNT3, KL, and SAMD9 pathogenic variants; however, results could not be confirmed as the patient was uncertain of his findings and hospital records for genetic testing were unavailable. Biopsy of soft tissue and bone showed fibrous tissue with focal dystrophic calcifications. Over nearly two decades, he developed large periarticular calcified masses and recurrent areas of skin compromise overlying some lesions. These episodes had previously required wound care and multiple incision and drainage procedures for presumed or confirmed superficial infection. Because chronic ulceration, drainage, scarring, and local inflammatory change were already part of his disease course, both the patient and treating clinicians were accustomed to interpreting new skin findings through a tumoral calcinosis framework.

On examination, temperature was 98.4°F, blood pressure 131/79 mmHg, heart rate 84 bpm, respiratory rate 16 breaths/min, and oxygen saturation 98% on room air, but he appeared fatigued. Musculoskeletal examination demonstrated firm, lobulated, noncompressible masses approximately 8 x 6 cm over the left knee region (Figure 1) and over the right elbow (Figure 2).

**Figure 1:**
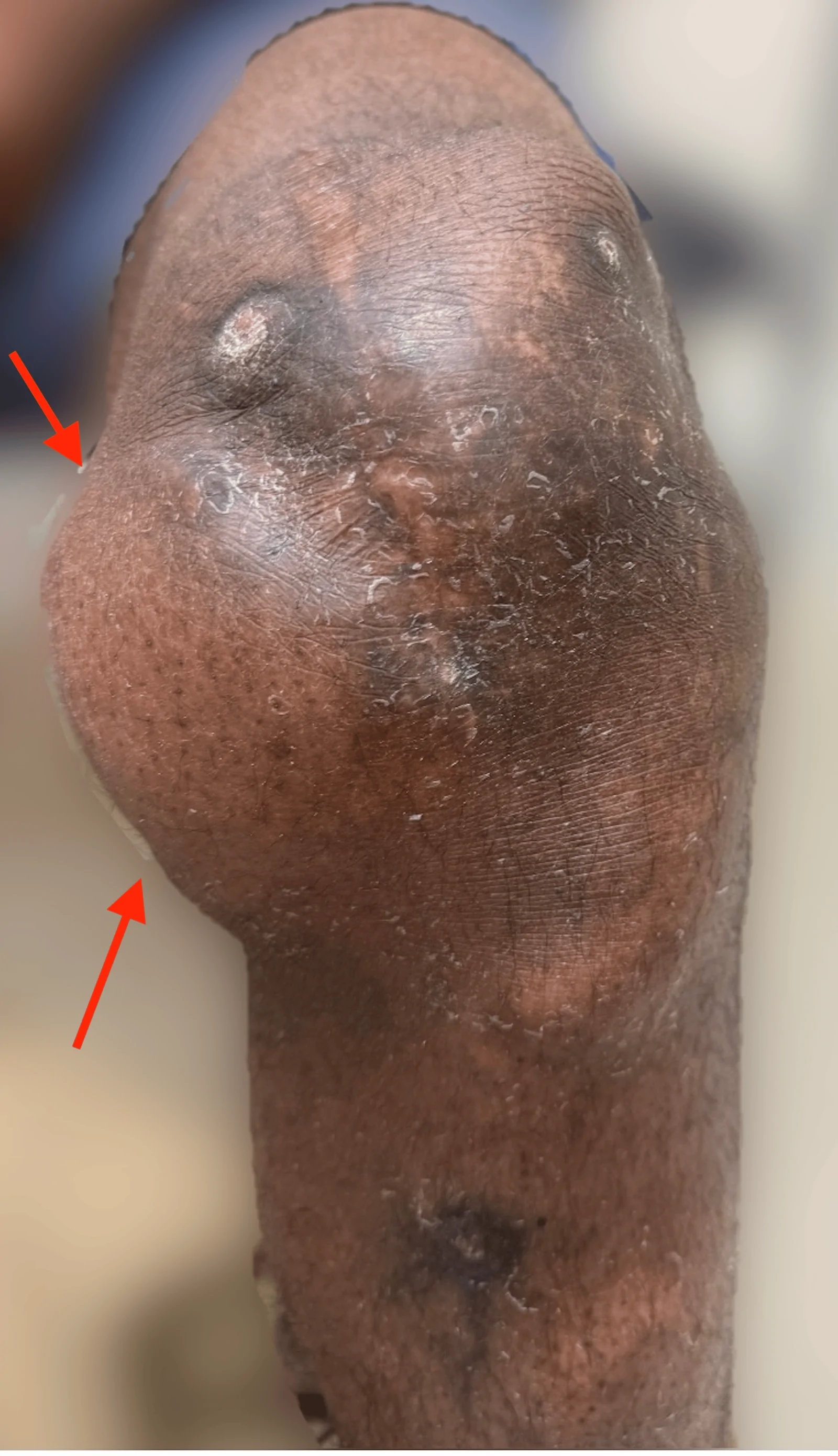
Firm, lobulated, noncompressible masses (red arrows) approximately 8 x 6 cm over the left knee region (red arrows)

**Figure 2:**
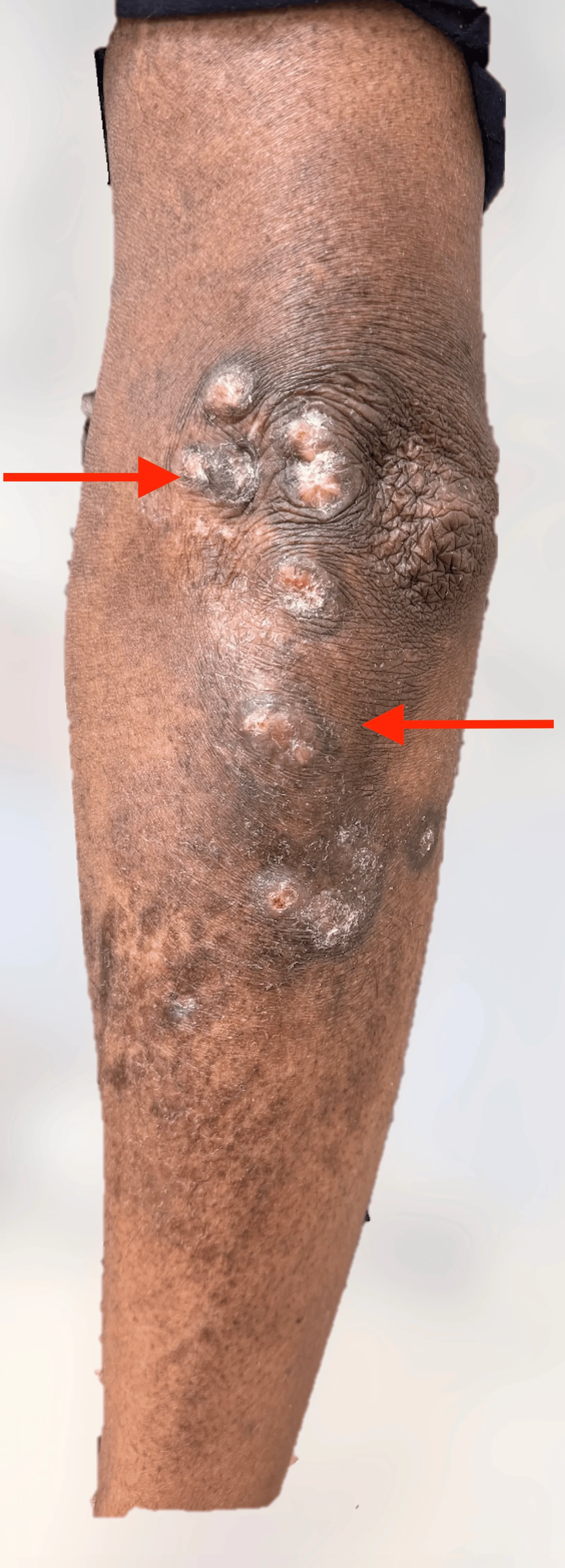
Left elbow with chronic scarring and small areas of serous discharge Red arrows point to multiple fibrous and healing ulcers that have resulted from recurrence flares of tumoral calcinosis.

The overlying skin showed chronic scarring and small areas of serous discharge without clear fluctuance, as seen in Figure 3. Left knee range of motion was limited at the extremes of flexion, consistent with the local mass effect of periarticular calcification. There was no obvious hot swollen joint to suggest acute septic arthritis. The axial skeleton was not tender. There was no documented focal neurologic deficit.

**Figure 3:**
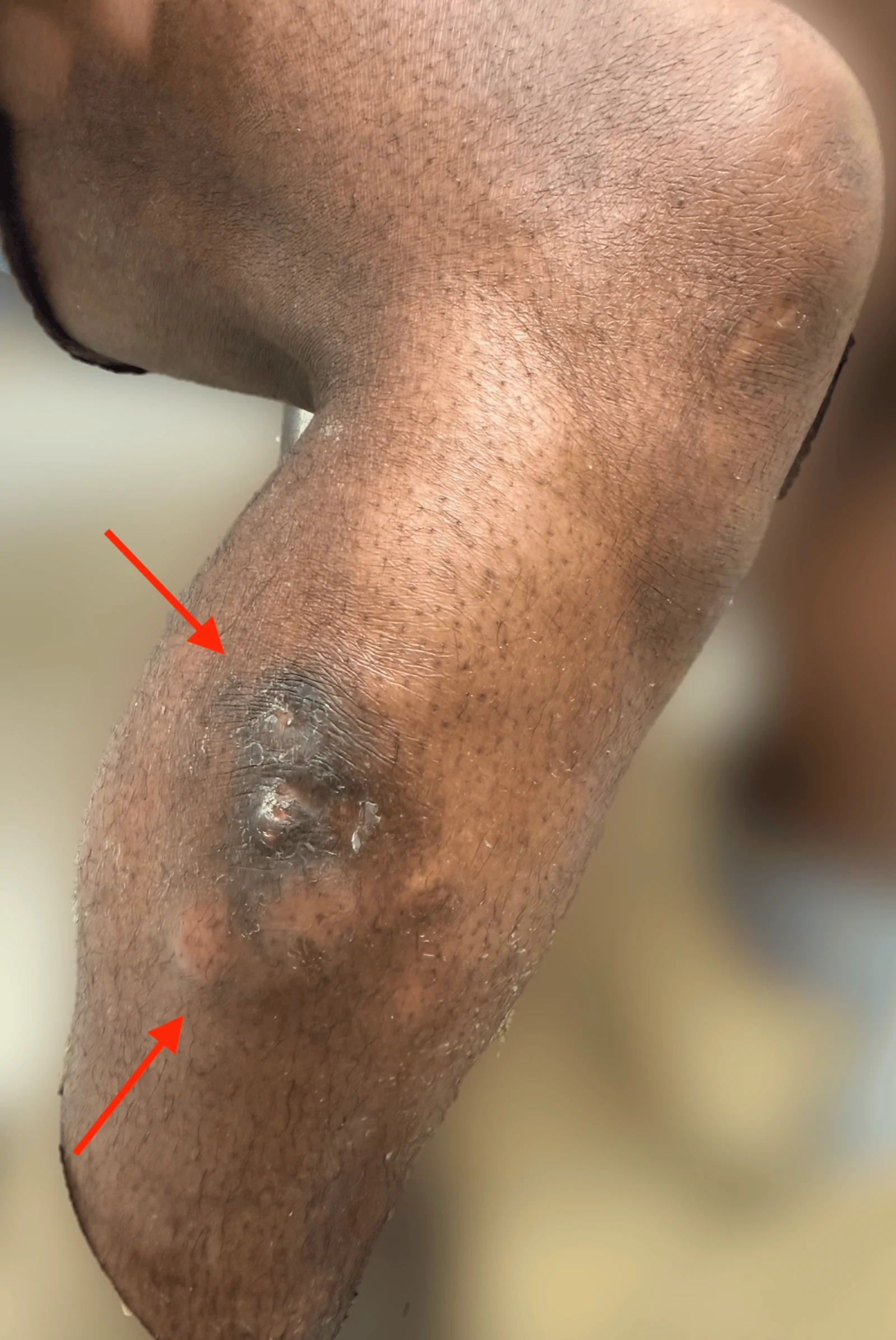
Recurrent tumoral calcinosis flares have resulted in multilobulated tumor-like masses on the lateral shin as identified by red arrows

The most important examination finding was cutaneous. Multiple painless, nonpruritic, shallow ulcerated, or eroded lesions were present on the palms (Figure 4) and dorsum of hands (Figure 5). The patient initially considered these lesions part of his general pattern of skin breakdown, and his earlier clinical encounters had not separated them from the chronic calcinosis narrative.

**Figure 4:**
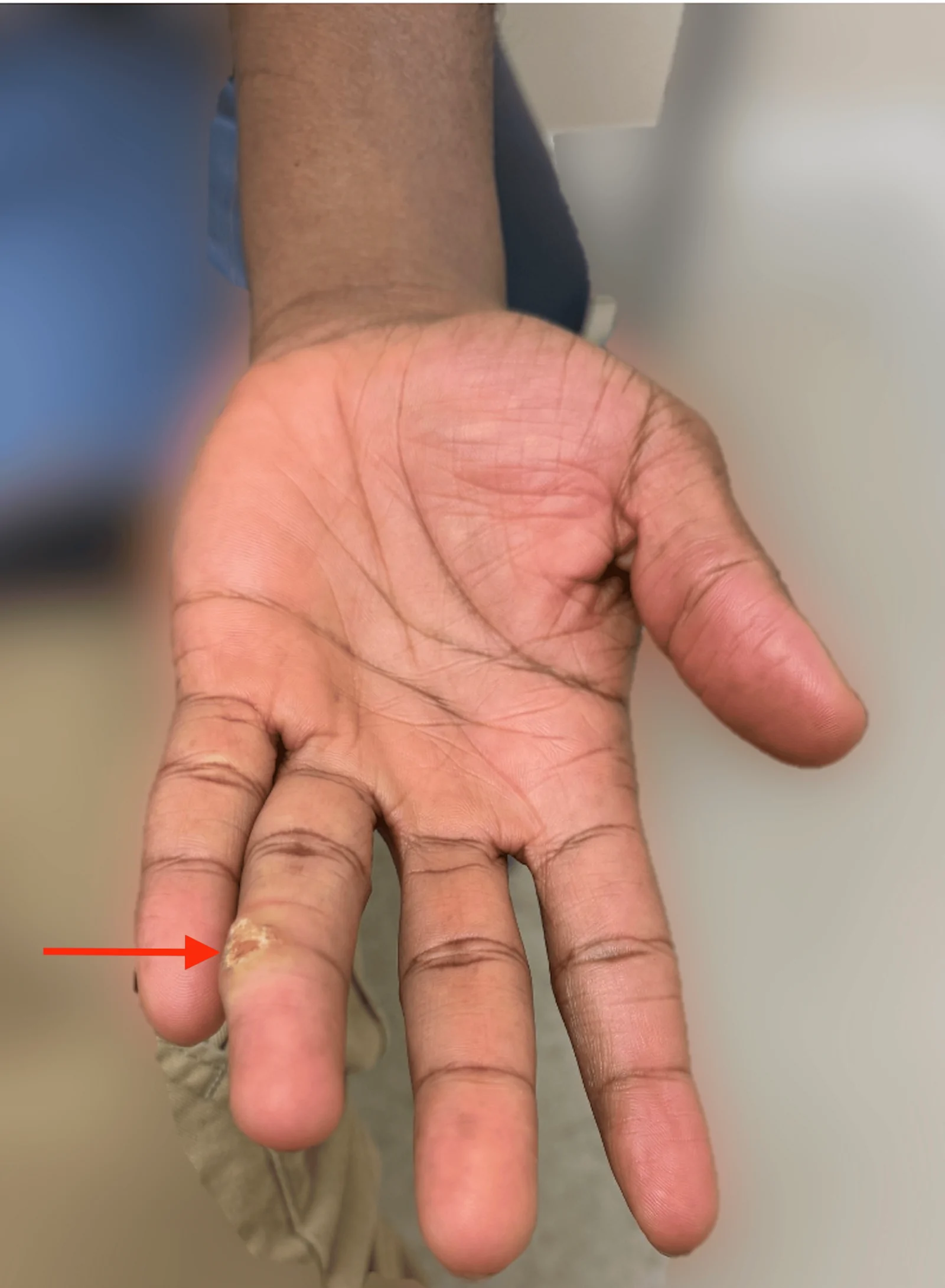
Red arrow shows ulcerated eroded lesion left fourth finger, likely sequelae of secondary syphilis

**Figure 5:**
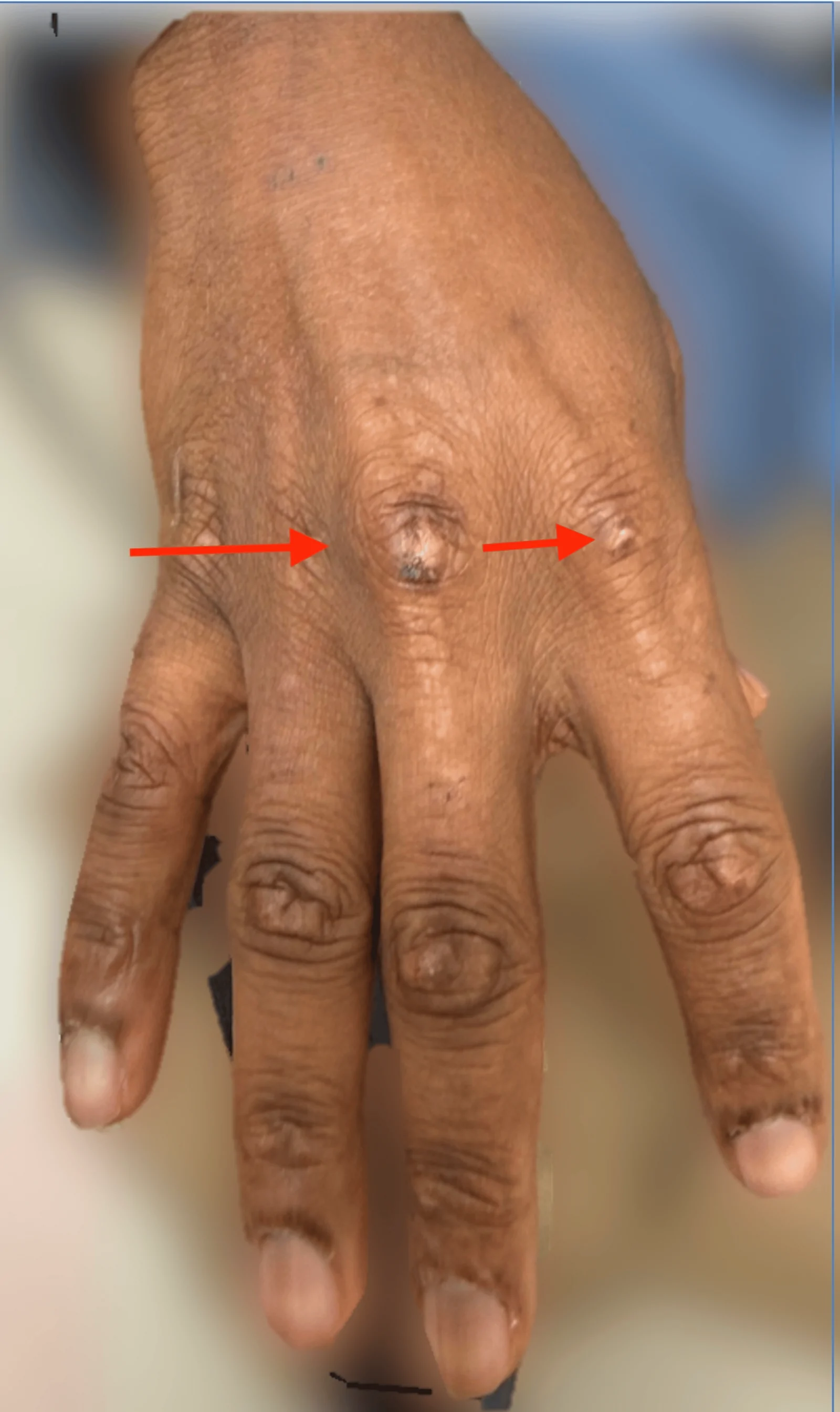
Red arrows show shallow ulcerated lesions on dorsum of the right hand, likely sequela of secondary syphilis

However, their palmar distribution, painless character, and association with fatigue and diffuse arthralgia were not typical for simple pressure-related ulceration over calcified periarticular masses. No genital ulcer or lymphadenopathy was reported and patient declined genital examination, although history later revealed a transient nonpruritic truncal rash several months earlier that had resolved spontaneously.

Initial laboratory studies showed normal serum phosphates of 3.2 mg/dL, with normal serum calcium and alkaline phosphatase. Renal function was preserved. Inflammatory markers were elevated: erythrocyte sedimentation rate (ESR) 80 mm/hr and C-reactive protein (CRP) 17 mg/L, supporting an inflammatory or infectious process but not distinguishing between a tumoral calcinosis flare, soft-tissue infection, systemic rheumatic disease, or a sexually transmitted infection. Vital signs, routine laboratory findings, and follow-up serology are summarized in Table 1.

**Table 1:** Vital signs and routine laboratory findings ESR = Erythrocyte Sedimentation Rate; CRP = C-Reactive Protein; RPR = Rapid Plasma Reagin; SpO_2_ = Peripheral Oxygen Saturation; BUN = Blood Urea Nitrogen; CO_2_ = Carbon Dioxide, eGFR = Estimated Glomerular Filtration Rate. Reference ranges reflect standard adult laboratory values.

Parameter	Result	Reference Range	Interpretation
Vital Signs			
Temperature	98.4°F (36.9°C)	97.8-99.1°F (36.5-37.3°C)	Normal
Blood Pressure	131/79 mmHg	<120/80 mmHg (normal)	Mildly elevated (systolic)
Heart Rate	84 beats per minute	60-100 beats per minute	Normal
Respiratory Rate	16 breaths per minute	12-20 breaths per minute	Normal
Oxygen Saturation (SpO_2_), on room air	98%	≥95%	Normal
Serum Chemistry			
Serum Phosphate	3.2 mg/dL	2.5-4.5 mg/dL	Normal
Serum Calcium	Within normal limits	8.5-10.2 mg/dL	Normal
Alkaline Phosphatase	Within normal limits	44-147 U/L	Normal
Creatinine	0.55 mg/dL	Serum creatinine: 0.74-1.35 mg/dL (male)	Slightly decreased
eGFR	>60.0 mL/min/1.73 m^2^	>60 mL/min/1.73 m^2^	Normal
Anion Gap	8 mmol/L	1-11 mmol/L	Normal
Sodium	138 mmol/L	136-145 mmol/L	Normal
Potassium	4.3 mmol/L	3.4-4.9 mmol/L	Normal
Chloride	100 mmol/L	98-107 mmol/L	Normal
CO_2_	30 mmol/L	21-32 mmol/L	Normal
Glucose	205 mg/dL	74-109 mg/dL	Elevated
Calcium	10.2 mg/dL	8.6-10.2 mg/dL	Normal
BUN	9 mg/dL	7-25 mg/dL	Normal
Inflammatory Markers			
Erythrocyte Sedimentation Rate (ESR)	80 mm/hr	0-15 mm/hr (male)	Elevated
C-Reactive Protein (CRP)	17 mg/L	<10 mg/L	Elevated
Serology			
Rapid Plasma Reagin (RPR), initial	Reactive; titer 1:32	Non-reactive	Positive
*Treponema pallidum* Antibody (confirmatory)	Reactive	Non-reactive	Positive
RPR, 3-month follow-up	Reactive; titer 1:8	Non-reactive	Fourfold decline; treatment response
Human Immunodeficiency Virus Antibody and Antigen	Non-reactive	Non-reactive	Normal
Trichomonas vaginalis	Not detected	Not detected	Normal
Neisseria gonorrhoeae	Not detected	Not detected	Normal
Chlamydia trachomatis	Not detected	Not detected	Normal
Herpes Simplex Virus Type 1	Not detected	Not detected	Normal
Herpes Simplex Virus Type 2	Not detected	Not detected	Normal

Radiographs and computed tomography (CT) of the ankle, as seen in Figures 6, 7, respectively, confirmed well-circumscribed, multiloculated periarticular calcifications in soft tissue, with a cloud-like appearance compatible with tumoral calcinosis. These imaging findings explained the local mass, chronic discomfort, and limited range of motion. They did not, however, adequately explain the diffuse fatigue, generalized arthralgia, or palmar lesions. The distinction was clinically important: imaging confirmed the chronic disease but did not prove that all current symptoms were caused by it.

**Figure 6:**
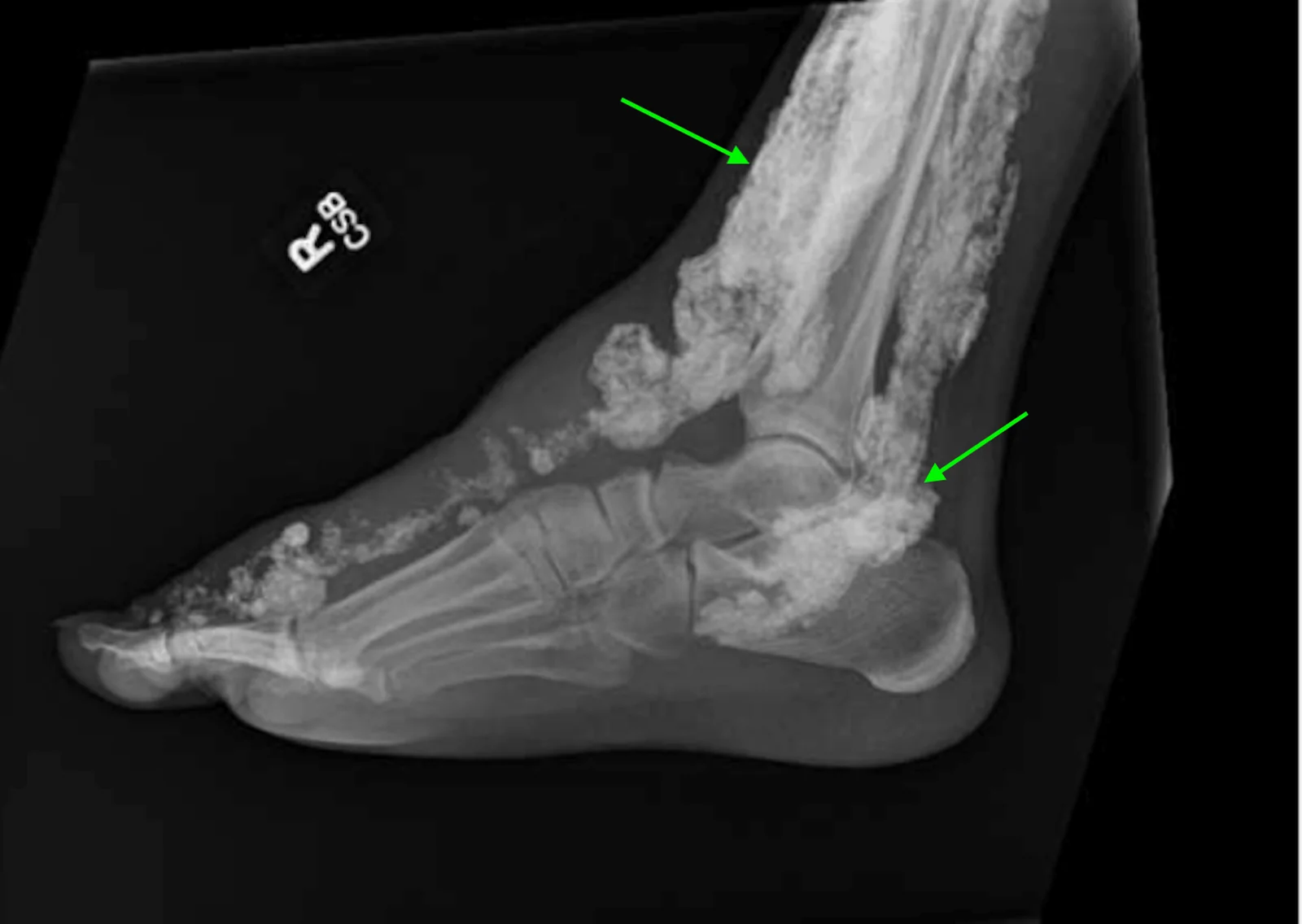
Lateral X-ray of the right ankle Green arrows point to extensive soft tissue calcification consistent with tumoral calcinosis over the right distal anterior calf, ankle, and dorsal aspect of the foot.

**Figure 7:**
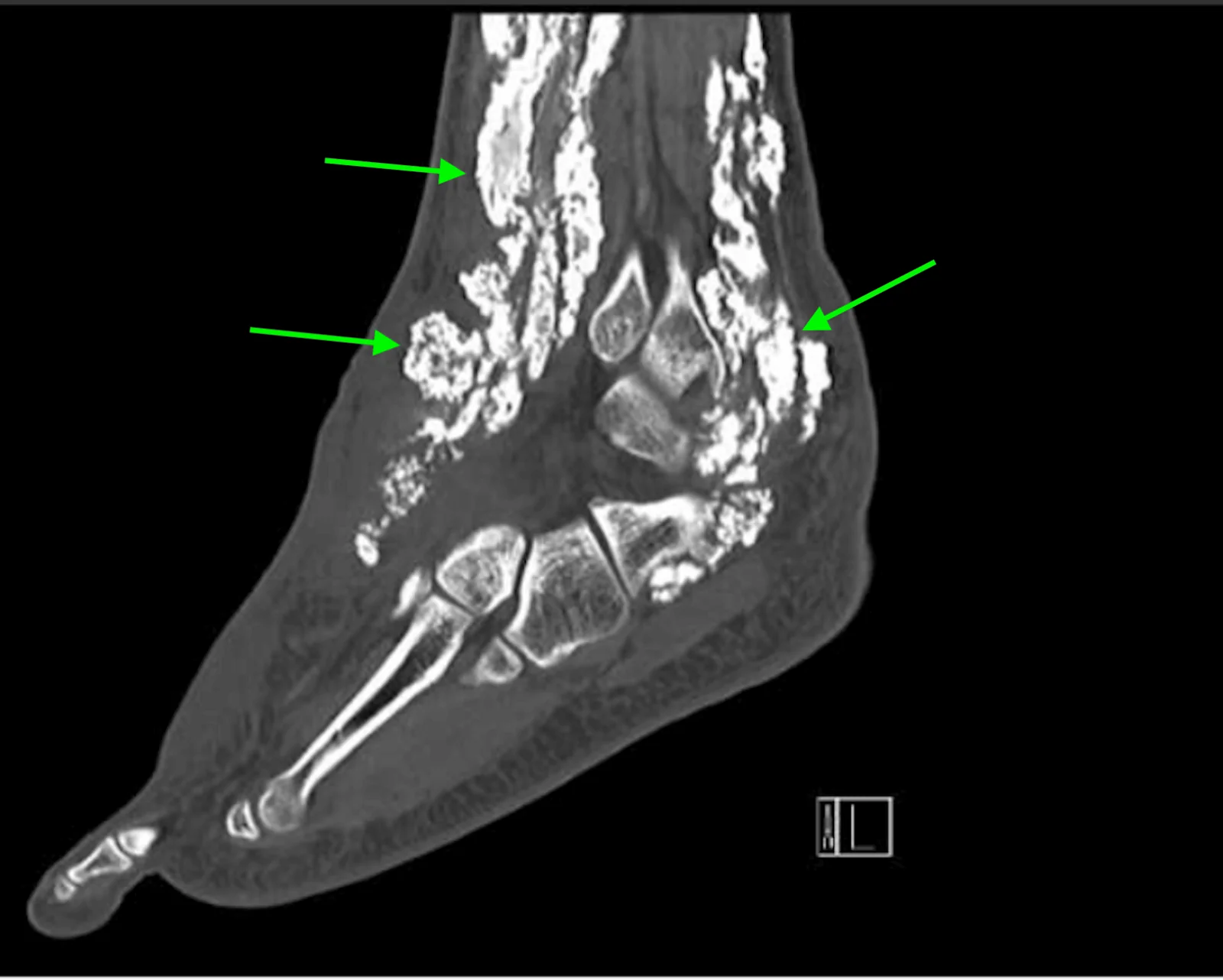
CT imaging of right ankle through foot showed stigmata of tumoral calcinosis Green arrows point to extensive soft tissue calcifications at the dorsal region of the right foot and right ankle as well as the distal anterior portion of the right calf.

Because the presentation contained systemic and dermatologic elements outside the expected local pattern of tumoral calcinosis, syphilis testing was performed. RPR test was positive with a titer of 1:32. Confirmatory *Treponema pallidum* antibody test was positive. After the positive serology, directed history revealed recent unprotected sexual encounters. The patient also recalled a transient, nonpruritic rash on the trunk approximately six months earlier, which had resolved without treatment. Taken together, the palmar lesions, constitutional symptoms, arthralgia, positive nontreponemal and treponemal testing, and timing of the prior rash supported a diagnosis of secondary syphilis with transition toward early latent syphilis.

The patient was treated with Doxycycline 100 mg twice daily for 14 days due to shortage of Penicillin G Benzathine which is consistent with recommended therapy for primary, secondary, and early latent syphilis in nonpregnant adults when neurosyphilis, ocular syphilis, or otosyphilis is not suspected. He was counseled on sexual transmission, abstaining from sexual activity until treatment was completed, partner evaluation and notification of recent partners, and the importance of follow-up serologic testing. Follow-up serology after three months showed decreased Rapid Plasma Reagin (RPR) titer from 1:32 to 1:8 representing an appropriate treatment response with fourfold decline in titer.

## Discussion

The main diagnostic lesson is the risk of anchoring bias. Anchoring occurs when the initial diagnostic impression receives disproportionate weight and subsequent information is interpreted to fit that impression. In this patient, tumoral calcinosis provided a highly visible and legitimate anchor. It was rare, longstanding, anatomically impressive, and known to cause pain, inflammation, ulceration, discharge, and infection. Yet the clinical question was not whether he had tumoral calcinosis; he clearly did. The question was whether tumoral calcinosis explained the full syndrome at this visit.

Diagnostic overshadowing is a closely related concept. It describes the tendency to attribute new symptoms to a pre-existing condition, especially when that condition is complex or unusual. Patients with rare diseases are particularly vulnerable because the rare diagnosis can dominate the medical record, referral pattern, and clinician attention. The more dramatic the chronic disease appears, the easier it is to miss a common condition occurring simultaneously. In this case, palmar lesions and systemic symptoms were at risk of being absorbed into the narrative of calcinosis-associated skin breakdown. Tumoral calcinosis can plausibly mimic infection or inflammation; periarticular ectopic calcifications can limit joint motion and cause inflammatory bony pain. Lesions may enlarge, ulcerate, drain hydroxyapatite-rich material, and become secondarily infected [5].

At the same time, the literature on syphilis increasingly reminds rheumatologists that *Treponema pallidum* can enter the musculoskeletal differential in many forms. Traczuk and colleagues reviewed adult musculoskeletal manifestations of syphilis and emphasized that early and late disease may present with articular and periarticular pathology, including inflammatory arthritis, tenosynovitis, periostitis, and myositis. Their report specifically asks what rheumatologists need to know because the clinical presentation may resemble autoimmune disease [6]. A 2025 Rheumatology clinical vignette by Durand and Lobbes similarly described secondary syphilis presenting as sternoclavicular arthropathy with erosions, inflammatory shoulder pain, elevated CRP, weight loss, aphthous stomatitis, unilateral hearing loss, and rash; inflammatory rheumatic serologies were negative, and symptoms resolved after penicillin therapy [7]. These recent reports align with the present case by showing that syphilis may present not just as a dermatologic or sexually transmitted infection, but as a systemic inflammatory syndrome entering the rheumatology clinic.

The mechanism of musculoskeletal syphilis is not fully captured by one pathway. Hematogenous dissemination during secondary disease can seed periosteum, bone marrow, synovium, tendon sheaths, and other connective tissues. Histology may show plasma cell-rich inflammation, vasculitic change, granulomatous reaction, or nonspecific synovitis. Imaging can reveal periostitis, osteitis, marrow edema, erosive-appearing arthropathy, or soft-tissue inflammation. Because these findings are not pathognomonic, they can be misclassified as autoimmune arthritis, chronic osteomyelitis, malignancy, or inflammatory bone disease. In a patient with tumoral calcinosis, this overlap is even more challenging because chronic foreign-body reaction to calcium deposits, recurrent ulceration, and secondary infection may produce inflammatory findings that resemble or conceal treponemal disease.

The skin findings were decisive. A rash involving the palms or soles is a classic clue to secondary syphilis and should prompt sexual history and serologic testing, particularly when accompanied by arthralgia, malaise, lymphadenopathy, mucous lesions, alopecia, hepatitis, nephrotic findings, ocular symptoms, or neurologic complaints. The lesions in this case were not simply overlying the elbow or knee calcific masses; they were on the palms, an anatomic site that did not match the patient's mechanical calcinosis burden. This bedside mismatch was the key to escaping the anchor. For rheumatologists, the habit of examining palms, soles, oral mucosa, scalp, nails, and genital or intertriginous areas when appropriate remains highly valuable. Inflammatory arthritis is rarely diagnosed by joint examination alone.

A second practical lesson concerns timing. The patient recalled a transient truncal rash six months earlier, suggesting that the window for recognizing secondary syphilis may have been missed. By presentation, the syndrome was interpreted as secondary syphilis transitioning toward early latent disease. Staging matters because treatment duration depends on stage. Centers for Disease Control and Prevention (CDC) guidance emphasizes that parenteral penicillin G is preferred for all stages and that the preparation, dose, and duration depend on clinical manifestations. For early syphilis, benzathine penicillin G 2.4 million units intramuscularly as a single dose is the standard therapy, but Doxycycline 100 mg orally two times a day for 14 days has been used for years and can be effective [8].

The case also sits within a changing epidemiologic landscape. CDC provisional 2024 surveillance data showed that total syphilis cases in the United States remained high despite a decrease from 2023, with 190,242 total syphilis cases reported in 2024 and nearly 4,000 congenital syphilis cases. Primary and secondary syphilis cases declined nationally for a second consecutive year, but the overall burden remains substantial and varies by geography and population. For clinicians, this means that syphilis should not be viewed as historical curiosity. It remains common enough to test for when compatible clinical clues are present and consequential enough to identify before initiating immunosuppression or invasive procedures [8,9].

From a journal perspective, the novelty of this case is not that syphilis can mimic rheumatic disease, that is established. The novelty is the masking environment created by tumoral calcinosis. Recent tumoral calcinosis publications continue to emphasize frequent misdiagnosis because of tumor-like masses, rarity, and complex imaging. A 2025 case report and literature review by Li and colleagues described tumoral calcinosis as a rare benign disorder of extraosseous calcification that is often misdiagnosed as tumor-related disease and emphasized the value of characteristic imaging in improving diagnostic accuracy [4]. A 2025 review by Donati and colleagues similarly summarized tumoral calcinosis as an extremely rare, disabling periarticular calcification disorder with limited treatment evidence [3]. The present case adds a different diagnostic problem: once tumoral calcinosis is correctly diagnosed, it can still become a cognitive screen through which unrelated infection is misread as chronic disease activity.

This case supports a structured approach to atypical flares in chronic rheumatologic or metabolic disease: define the patient’s usual flare pattern; identify new or discordant features; determine whether findings localize to the known disease; screen for common mimics before escalating disease-specific therapy; and revisit the diagnosis when new data emerges. This approach is especially important before immunosuppression. Although this patient was not described as receiving glucocorticoids, biologics, or conventional disease-modifying antirheumatic drugs, many patients presenting to rheumatology with arthralgia, rash, elevated inflammatory markers, or erosive-appearing lesions may receive anti-inflammatory or immunosuppressive therapy if infection is not considered. Treating an infectious mimic as autoimmune disease can delay definitive therapy, increase transmission risk in the case of syphilis, and expose the patient to unnecessary harm. Conversely, prompt serologic testing is inexpensive, accessible, and clinically decisive when pretest probability is raised by rash or exposure history.

This case report has several limitations, including absence of histopathology of the palmar lesions. Dark-field microscopy and polymerase chain reaction (PCR) testing were not performed. Detailed inflammatory marker values and cerebrospinal fluid (CSF) evaluation were not available. The diagnosis was based on a compatible clinical syndrome, positive RPR titer, and treponemal antibody testing, which is clinically appropriate in most settings. Precise staging as late secondary syphilis versus transition to early latent syphilis is limited by timeline details and public health staging definitions. Despite these limitations, the key teaching point remains strong: a confirmed diagnosis of tumoral calcinosis did not exclude concurrent syphilis.

## Conclusions

This case demonstrates that a rare chronic diagnosis can be both clinically real and diagnostically distracting. Tumoral calcinosis explained the patient's longstanding periarticular masses, local discomfort, limited elbow motion, scarring, and recurrent skin complications. It did not fully explain painless palmar lesions, diffuse fatigue, generalized arthralgia, compatible sexual exposure history, and reactive syphilis serology. Recognition of this mismatch allowed diagnosis and treatment of syphilis.

For rheumatologists, the case reinforces three practical messages. First, atypical flares deserve a fresh differential diagnosis rather than automatic attribution to the established disease. Second, infection screening should be guided by symptoms, anatomy, and exposure history, not by whether another diagnosis is already present. Third, syphilis remains a relevant and treatable mimic of rheumatic disease. A careful skin examination, including palms and soles, and a willingness to ask a targeted sexual history can prevent unnecessary procedures, delayed antimicrobial therapy, and inappropriate immunosuppression.
